# Hwangryunhaedoktang in adult patients with Atopic Dermatitis: a randomised, double-blind, placebo-controlled, two-centre trial - study protocol

**DOI:** 10.1186/1472-6882-11-68

**Published:** 2011-08-23

**Authors:** Nam-Kwen Kim, Dong-Hyo Lee, Hyung-Sik Seo, Seung-Ho Sun, Yong-Leol Oh, Ji-Eun Kim, In-Hwan Yoon, Eun-Sung Seo, Gye-Seon Shim, Christopher Zaslawski

**Affiliations:** 1 Opthalmology Otolaryngology & Dermatology, Wonkwang Univ. Sanbon Oriental Medical Center, Gunpo, Korea; 2 Opthalmology Otolaryngology & Dermatology, Pusan National Univ. School of Korean Medicine, Yangsan, Korea; 3 Internal Medicine, Sangji Univ. Oriental Medical Center, Wonju, Korea; 4 Gastroenterology, Wonkwang Univ. Sanbon Medical Center, Gunpo, Korea; 5 Opthalmology Otolaryngology & Dermatology, Sangji Univ. Oriental Medical Center, Wonju, Korea; 6 Department of Oriental Medicine, Graduate School of Wonkwang University, Iksan, Korea; 7 College of Traditional Chinese Medicine, Department of Medical and Molecular Biosciences, Faculty of Science, University of Technology, Sydney, Australia

## Abstract

**Background:**

Atopic Dermatitis is a chronic relapsing eczematous skin disease with increasing prevalence and rising costs. It has a clear impact on a patient's quality of life. Many patients are worried about the use of usual care techniques, such as corticosteroids and antihistamine due to the widespread fear of adverse effects. Complementary and alternative medical approaches have been employed to relieve symptoms of Atopic Dermatitis. Hwangryunhaedoktang is among the most strongly preferred and widely used herbal medicines for Atopic Dermatitis in Korea, as it causes very few serious adverse effects.

We aim to establish basic clinical efficacy and safety data for Hwangryunhaedoktang, which is approved as an herbal medication by the Korean Food and Drug Administration, in adult patients with Atopic Dermatitis.

**Methods/Designs:**

This study is a randomised, double blind, placebo-controlled, two-centre trial with two parallel arms (Hwangryunhaedoktang and a placebo). The diagnosis of Atopic Dermatitis will be made according to the criteria of Hanifin and Rajka by two different Oriental medicine doctors. We will include participants experiencing typical conditions of intermittent or continuous Atopic Eczema for six or more months. Participants will receive Hwangryunhaedoktang or a placebo-drug for eight weeks. The total duration of each arm is eleven weeks. Each participant will be examined for signs and symptoms of Atopic Dermatitis before and after taking medication. A follow-up to evaluate the maintenance of safety will be performed two weeks after the final administration of medication.

**Discussion:**

This trial will utilize high quality trial methodologies in accordance with consolidated standards of reporting trials guidelines. It will provide evidence for the clinical efficacy and safety evaluation of Hwangryunhaedoktang in adult patients with Atopic Dermatitis. Moreover, we will also employ health-related quality of life questionnaires to assess the changes in quality of life.

**Trial registration:**

Current Controlled Trials ISRCTN26218532

## Background

Atopic Dermatitis (AD) is a chronic inflammatory pruritic skin disease that is often associated with other atopic disorders such as allergic rhinitis and asthma[1]. It is characterised by poorly defined erythema with oedema, vesiculation, and weeping in the acute stage and skin thickening (lichenification) in the chronic stage[2]. These irritating symptoms result in a severely reduced quality of life (QOL). In particular, itching that continues throughout the day and worsens at night causes sleep loss and impacts everyday activities and psychosocial well being [3,4].

The prevalence of AD has doubled or tripled in industrialised countries during the past three decades; 15% to 30% of children and 2% to 10% of adults are affected[5]. Childhood disease continues into adulthood in a significant number of individuals and represents a notable burden[6]. AD should be regarded as a condition that has the potential to be a major handicap involving considerable personal, social and financial consequences both for the family and for the community[7].

The most common therapies for the treatment of moderate and severely relapsing AD are topical corticosteroids, emollients, oral antihistamines, antibiotic agents, immunosuppressive agents, or non-pharmacologic approaches[2]. However, these methods do not ensure successful treatment in every patient with AD. Oral corticosteroids improve the lesions of AD, but a disease flare may occur when these medications are stopped. In addition, the local and systemic side effects of topical steroids, such as skin atrophy, hypo-pigmentation, adrenal suppression, cataracts, glaucoma, and growth retardation, are well recognised[8]. Although pruritus can be treated with antihistamines or tricyclic anti-depressants, these agents can affect a child's ability to learn or an adult's ability to drive and work[9].

In this context, various complementary and alternative medicine (CAM) treatments have been administered for AD in clinical practice. Complementary and alternative therapies are commonly used because of concerns about the potentially adverse effects of conventional therapies and frustration with the lack of appropriate regular use[10]. Some studies have reported the effectiveness of acupuncture or herbal medicine, which is the most popular form of CAM therapy, for the treatment of AD when used alone or concomitantly with usual care[10-12]. In the few number of trials that have been conducted, the quality and the small sample sizes of these studies make it difficult to reach firm conclusions concerning these treatments. Well-designed, randomised, controlled trials are needed to examine the efficacy of complementary and alternative treatments for AD.

The purpose of this study is to establish the basic clinical efficacy and safety data for Hwangryunhaedoktang, which is approved as an herbal medication by the Korean Food and Drug Administration (KFDA). The use of Hwangryunhaedoktang for AD is based on the principles of Traditional Korean Medicine (TKM). In this medical tradition, Hwangryunhaedoktang is administered to treat the pattern of wind-heat, blood-heat, or blood deficiency associated with AD[13]. Also, several studies demonstrated that Hwangryunhaedoktang exerts anti-inflammatory, antibiotic, antioxidative, and immunosuppressive activities[14,15]. However, there are no relevant randomised, controlled clinical trials regarding AD.

We will conduct a randomised, double-blind, placebo-controlled, two-centre trial of Hwangryunhaedoktang in adult patients with AD. Because of the absence of a gold standard for the treatment of AD, this trial will be conducted to determine the effectiveness of these herbal medicines by comparison with a placebo controlled group.

## Methods/Design

**Figure 1:**
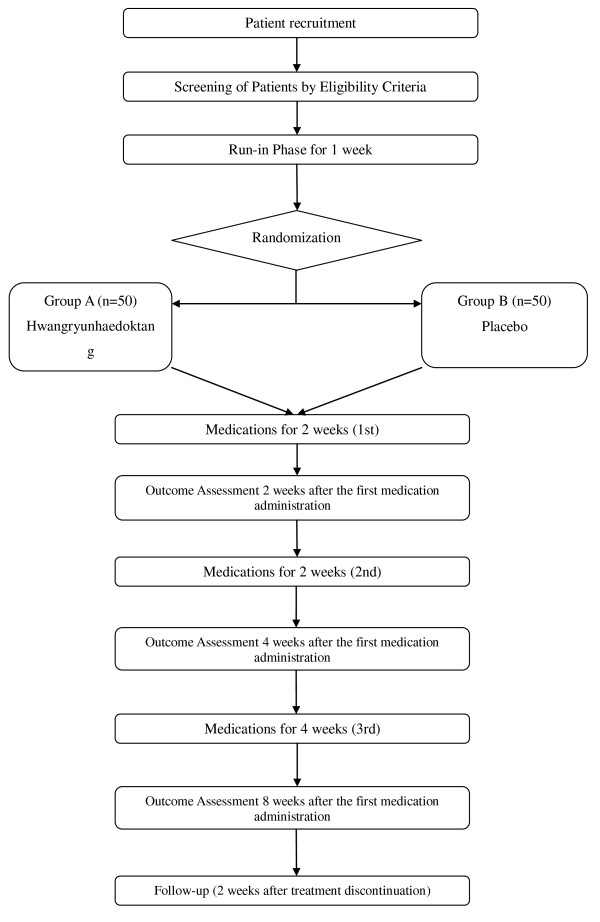
**Study Flow Chart**.

A randomised, double-blind, placebo-controlled, two-centre trial will be conducted at the Wonkwang University Oriental Medical Centre in Gunpo and at the Sangji University Oriental Medical Centre in Wonju, Korea. Participants fulfilling eligibility criteria will be selected. Enrolled participants will be randomly allocated to two parallel groups: the Hwangryunhaedoktang and placebo arms. Each participant will be examined for signs and symptoms of AD before and after taking medication. A follow-up to evaluate the maintenance of safety will be performed by phone-call (Figure 1).

### Recruitment

We will recruit participants by advertisement in local newspapers and on the websites of local medical centres in Gyeonggi-province and Gangwon-province. Respondents will be contacted by clinical trial coordinators (CRC) to determine eligibility via a telephone pre-screen. If an applicant meets the study criteria, he or she will be invited to the clinical research centres and be examined for eligibility. Oriental medicine doctors and Internal medicine doctors will conduct the screening together to improve the quality of eligibility assessment. Oriental medicine doctors will manage the whole process of screening. Internal medicine doctors will assess the complete blood cell count (CBC), erythrocyte sedimentation rate (ESR), blood chemistry, PA chest film, and urine analysis to exclude patients with serious liver and kidney disease.

### Inclusion criteria

Eligible participants of both sexes who are 19 years or older will be enrolled according to the inclusion and exclusion criteria. The diagnosis of AD will be made according to the criteria of Hanifin and Rajka by two different Oriental medicine doctors. We will include participants experiencing the typical conditions of intermittent or continuous Atopic Eczema for six or more months. Participants will be instructed to discontinue other treatments for AD during the run-in, treatment, and follow-up periods. Written informed consent will be obtained from each participant.

### Exclusion criteria

Participants will be excluded if they suffer from serious medical conditions, such as uncontrolled hypertension, diabetes mellitus requiring insulin injection, past or current malignancy, liver or kidney dysfunction, anaemia, active pulmonary tuberculosis, or other severe dermatitis or infectious or systemic diseases incompatible with treatment with herbal medicines. The exclusion criteria are as follows: administration of steroids and immunosuppressants *per os *within one week of interview; the use of other forms of AD treatment except those involving external application; pregnancy, lactation, or the lack of a form of contraception; underlying disease or history of severe hepatic disease, abnormal results for liver function test (greater than twice the upper limit of normal); participation in another clinical trial within one month of enrolment; hypersensitivity or allergic reactions to herbal medicines that are related to this trial; diseases that can affect the absorption of drugs or disordered digestion after surgery related to such a disease; inability to understand written consent or engage in this study due to mental retardation or other emotional or mental problems; or judgment by experts that the potential subject's participation is inappropriate.

Exclusion will be primarily based on the information provided by the patients. Also, additional examinations, such as CBC, ESR, blood chemistry, PA chest film, urine analysis, and vital signs will be performed before the trial to ensure that the patients do not suffer from these diseases. Over-the-counter (OTC) drugs will be allowed for the management of headaches, dyspepsia, etc.; however, participants will be gently advised to reduce the doses, stop taking supplements, or refrain from adding other supplements to their regimen. Some OTC drugs, such as those containing anti-histamine or anti-allergenic agents, will not be allowed, as there are concerns that these drugs might have direct impacts on AD.

These restrictions will be based on the information provided by patients, and researchers will then evaluate whether or not some of these components would impact AD. We will record the drugs taken by each participant at every visit, and participants will be asked to notify us of any changes in their medication/supplement regimen. Additional herbal prescriptions, acupuncture treatments, or therapeutic interventions by other clinicians will not be allowed during the trial period. The complete eligibility criteria are in Appendix 1.

### Sample size

We wished to estimate the sample size that would be sufficient to detect significant differences in the SCORing Atopic Dermatitis (SCORAD) index between the experimental and control groups. Sample size was determined prospectively, as described in a previous study[16,17]. The mean and standard deviation of expected difference scores in SCORAD were 13 and 20 between the experimental and control group. The following formula was used to estimate the sample size for a two-group trial:

$$\frac{2{\left(Z_{\alpha ∕2}+Z_{\beta }\right)}^{2}\sigma^{2}}{{\left(\mu_{c}-\mu_{t}\right)}^{2}}$$

Calculations were performed using 80% power, a 5% significance level, and a 25% dropout rate. The required sample size was approximately 37 participants for each group. We plan to enrol 100 participants in each of the two groups, allowing for a 25% withdrawal rate.

### Randomisation and allocation

The randomisation procedure will be guaranteed by the authorised Clinical Research Organisation (CRO). An expert on statistics from CRO who have no contact with the study participants will conduct the randomisation of participants. Participants will be assigned random numbers via random number generator program (Microsoft Office Excel 2007). Random numbers will be sent to each centre, and randomisation table will be kept blinding by CRO during the research period. Ultimately, 100 participants will be randomised into the two groups at the two centres. However, the number of subjects planned for each location is not fixed. Opaque sealed envelopes containing serial numbers will be delivered to each centre. Opaque sealed randomisation table kept by CRO should be opened as a Standard Operating Procedure (SOP). If it has to be opened in urgent situation dealing with serious adverse events, primary investigator must report that to the CRO. Before the random assignment, all participants will be informed that they would be allocated to one of two groups. Random allocation will be conducted at the second visit. Random numbers with their corresponding participants will be determined by the order of the time of second visit. The allocation results will not be announced to the participants until the last visit of the last randomised participant.

### Blinding

In this trial, investigators will not be in contact with the CRC, the clinical pharmacist, or the statistician. The success of blinding will be assessed at each participant's last visit. Researchers who are blinded to the allocation results will perform the outcome assessment. The blinding procedure will also be verified by the authorised CRO.

### Treatment protocol

Participants will receive Hwangryunhaedoktang or a placebo-drug for eight weeks. Oral administration occurs according to the following statement:

Patients in group 1 will receive Hwangryunhaedoktang and instructions on how to make the tea; they should consume a packet of the medicine (5.00 g) with tepid water three times a day 30 minutes after meals.

Patients in group 2 will receive the placebo medicine (powdered extract), used in the same way as in group 1.

The defined daily doses (DDDs) of these herbal medicines should be determined based on the specifications and analytical procedures of drug products in KFDA guidelines. However, no pilot study has been reported so far.

#### Experimental medicines (Hwangryunhaedoktang)

Hanpoong Pharm and Foods Co., Ltd., produced the Hwangryunhaedoktang and the placebo medicine according to Korea Good Manufacturing Practice (KGMP) standards.

Hwangryunhaedoktang used in this trial is a brown, bitter herbal extract. Hwangryunhaedoktang is composed of four herbs: Powdered extract of Scutellaria baicalensis (334.00 mg as Baicalin), 1.67 g; Powdered extract of Gardenia jasminoides, 1.67 g; Powdered extract of Coptis chinensis (116.20 mg as Berberine), 0.83 g; Powdered extract of Phellodendron Amurense (19.92 mg as Berberine), 0.83 g.

#### Placebo medicine

Hanpoong Pharm and Foods Co., Ltd., developed a homogenous powdered extract of brown colour, which was made by mixing 3.489 g of lactose, 1.495 g of starch, and 0.016 g of pigments approved by KFDA. The colour, form, weight, odour, and taste of the placebo are very similar to the treatment medicines.

### Primary outcome measurement

SCORAD index will be used as the primary outcome variable. SCORAD is a clinical tool for assessing the severity of AD as objectively as possible. SCORAD consists of three scales: extent, intensity, and subjective. The severity of eruptions on lesions is evaluated on a 4-point scale (0: none, 1: mild, 2: moderate, 3: severe) by scoring the erythema, oedema, oozing, excoriation, lichenification, and xerosis. SCORAD is calculated by the equation A/5 + 7 × B/2 + C. In this equation, A is the percentage of body area with a skin rash, B is the total score of erythema, oedema, oozing, excoriation, lichenification, and xerosis, each of which was evaluated on a 4-point scale, and C is the visual analogue scale (VAS, a scale from 0 to 10) for subjective assessment of both itching and sleep loss[18-20]. It will be measured at treatment initiation, at two weeks after the first medication administration, at four weeks after the first medication administration, and at eight weeks after the first medication administration.

### Secondary outcome measurement

Secondary outcome measurements include the total IgE and eosinophil counts. These will be measured at treatment initiation, at four weeks after the first medication administration, and at eight weeks after the first medication administration. The EuroQoL 5-Dimension (EQ-5D), the Health Utilities Index Mark 3 (HUI3), and the Dermatology Life Quality Index (DLQI) will be measured at treatment initiation and at eight weeks after the first medication administration, as a measure of health outcomes. The data collection schedule is detailed in Table 1.

**Table 1:** Data Collection Schedule

Period	Screening	Treatment period				Followup
**Visit**	**1st** **(D -7)**	**2nd** **(D 1)**	**3rd** **(D 15)**	**4th** **(D 29)**	**5th** **(D 57)**	**(D 71)**

Informed consent	◯					

Demographic characteristics	◯					

Medical History	◯					

Safety Assessment	◯	◯	◯	◯	◯	

Total IgE, Eosinophil count		◯		◯	◯	

SCORAD Index		◯	◯	◯	◯	

Dermatology Life Quality Index		◯			◯	

EQ-5D		◯			◯	

Health Utilities Index Mark 3		◯			◯	

Adverse effects			◯	◯	◯	◯

Blinding Assessment					◯	

### Patient safety

All patients will undergo routine testing that included the following: complete blood cell count (CBC), erythrocyte sedimentation rate (ESR), and blood chemistry, as well as PA chest film and urine analysis before randomisation and immediately after completing the treatment. Vital signs will be measured at every visit. These tests help to identify and exclude patients with serious liver and kidney disease as well as those with other severe illness. Investigators will assess a significant result in these tests according to explicit criteria defining the incidence and intensity of adverse events. Also a causality evaluation of adverse drug reactions will be discussed.

### Statistical analysis

Analyses will be performed for two populations: 1) an intention-to-treat population consisting of all randomised participants who have at least one measurable outcome report following treatment (missing data are replaced with the last observation values) and 2) a per-protocol population including only participants without major protocol deviations. All data will be descriptively analysed. All main analyses will be based on the intention-to-treat population and conducted using LOCF (last observation carried forward) imputation method. For primary outcome measures, the mean differences from baseline values to the end of treatment will be compared using a independent T-test (Expected noncentrality parameter = 2.87, Critical t = 1.99, Power = 0.80). Paired T-test or non-centrality Wilcoxon rank sum test will be used for comparing safety variables. Repeated measure ANOVA (analysis of covariance) or repeated measure ANCOVA tests will also be performed for within-group and between-group analyses. Baseline data will be collected on each participant at randomisation in trial can be used to describe the population of patients, to assess comparability of treatment groups, to achieve balanced randomisation, to adjust possible comfoundings for prognostic factors, and to undertake subgroup analyses. We will exercise caution when drawing conclusions from subgroup findings.

Statistical analyses will be performed using the STATA statistical package program (ver. 11.0), and the level of significance is established at α = 0.05.

### Data and safety monitoring

To maintain the quality of this trial, monitoring will be conducted by Pharma CRO, a CRO located in Gyeonggi, Korea. Investigators can also be convened to discuss practical issues that might be encountered, such as dealing with serious adverse events, revising the protocol, and addressing certain important issues that might be raised by investigators and participants.

We defined adverse events as unintended signs, symptoms, or diseases occurring after treatment that were not necessarily related to the intervention. The safety assessment is based primarily on the frequency of adverse events, which include all serious adverse events. Information regarding adverse events will be summarised by presenting the number and percentage of participants experiencing any adverse events. During the trial, all adverse events will be observed in detail and documented in case report forms (CRFs).

### Ethics

Written consent will be obtained from each participant. This study protocol was approved by the institutional review boards (IRB) of the Wonkwang University Oriental Medical Centre, Sangji University Oriental Medical Centre, and Wonkwang University Medical Centre.

## Discussion

Herbal products have gained increasing popularity, and are now used by approximately 20% of the population in the United States[21]. But, use of botanicals is still relatively small compared to use of mainstream medicine in this continent. In contrast, the situation in Korea could not be more different[22]. Almost 60% of the total population had the experience of being treated by TKM[23]. Hwangryunhaedoktang is widely used herbal medicines for AD in Korea. There is a high possibility that some participants would already have an experience of being treated by Hwangryunhaedoktang. This trial will be conducted to assess the effectiveness of Hwangryunhaedoktang by comparison with a placebo controlled group. If participants would discriminate between placebo and experimental medicines during the treatment period, results of this trial may be affected by selection and detection bias. In order to prevent this situation, we developed a homogenous powdered extract. The colour, form, weight, odour, and taste of the placebo are very similar to the treatment medicines, Hwangryunhaedoktang. We will assess the success of blinding at each participant's last visit.

Most CAM trials do not describe the generation of the random sequence, an adequate method of allocation concealment, and the number and reasons for drop outs and withdrawals[24,25]. In this context, there are two unique features in this trial. Firstly, we developed the protocol in conjunction with the recommendations of consolidated standards of reporting trials (CONSORT) checklists for herbal interventions. Secondly, we adapted outcome measurements from prior studies on the herbal medicines for AD after reviewing the related research literatures.

This protocol is approved as an Investigational New Drug Application (IND) by the KFDA. This trial will provide for the clinical efficacy and safety evaluation of Hwangryunhaedoktang, herbal extracts, in adult patients with AD. We will also estimate the QOL changes from health-related quality of life questionnaires. Results of this trial will provide important information, in addition to addressing the feasibility of conducting a larger study using this methodology and statistical approaches as described in the Methods/Design section. Furthermore, it could provide a foundation for the understanding of health economic analysis.

## Abbreviation

AD: Atopic Dermatitis; ANOVA: analysis of variance; ANCOVA: analysis of covariance; CAM: complementary and alternative medicine; CBC: complete blood cell count; CONSORT: Consolidated Standards of Reporting Trials; CRC: clinical trial coordinators; CRFs: case report forms; CRO: Clinical Research Organisation; DDDs: defined daily doses; DLQI: Dermatology Life Quality Index; ESR: erythrocyte sedimentation rate; EQ-5D: EuroQoL 5-Dimension; KFDA: Korean Food and Drug Administration; HUI3: Health Utilities Index Mark 3; IND: Investigational New Drug Application; IRB: institutional review board; KGMP: Korean Good Manufacturing Practice; KHIDI: Korean Health Industry Development Institute; LOCF: Last Observation Carried Forward; OTC: Over the counter; QOL: Quality of Life; SCORAD: SCORing Atopic Dermatitis; SOP: Standard Operating Procedure; TKM: Traditional Korean Medicine; VAS: Visual Analogue Scale.

## Competing interests

The authors declare that they have no competing interests.

## Authors' contributions

All authors participated in the conception and design of the trial. NKK is the principal investigator of this study. He drafted the protocol. NKK and DHL wrote the final manuscript. HSS, SHS, YLO, JEK, IHY, ESS, GSS, CZ and DHL contributed to the research design and made critical revisions. All authors read and approved the final manuscript.

## Appendix 1. Eligibility Criteria

Inclusion criteria

1. Age greater than 19 years, either sex

2. Typical conditions of intermittent or continuous atopic eczema

2.1. Duration of more than 6 months

2.2. Satisfied Hanifin and Rajka's criteria for atopic dermatitis

3. Diagnosed with adult atopic dermatitis by two different oriental medicine doctors

4. Written and informed consent

Exclusion criteria

1. Other dermatitis or systemic disease except for atopic eczema

2. Administration of steroids and immunosuppressant *per os *(by mouth) within one week of the interview (topical application not relevant)

3. Women who are pregnant, lactating, or without contraception

4. Clinical severe hepatic disease or abnormal liver function tests at least twice the upper limit of normal

5. Participation in another clinical trial within the last month

6. Hypersensitivity or allergy to drugs

7. Disease which can affect the absorption of drugs, or disordered digestion after surgery related to the disease

8. Cannot understand written consent or follow this study

8.1. Mental retardation

8.2. Mental or emotional problems

9. Judged by expert as inappropriate to participate in this study

## Pre-publication history

The pre-publication history for this paper can be accessed here:

http://www.biomedcentral.com/1472-6882/11/68/prepub
